# Association between Ambient Ultrafine Particles and Neurodevelopmental Delay in Preschoolers in Shanghai, China

**DOI:** 10.1021/envhealth.4c00102

**Published:** 2024-10-01

**Authors:** Mengxun Rong, Yang Shen, Yihui Ge, Wenchong Du, Haidong Kan, Jing Cai, Yan Zhao, Jing Hua

**Affiliations:** † Department of Environmental Health, School of Public Health, Fudan University, 130 Dong-An Road, Shanghai 200032, China; ‡ Department of Psychology, Nottingham Trent University, Burton Street, Nottingham NG1 4BU, U.K.; § Hospital of Obstetrics and Gynecology, Shanghai Medical School, Fudan University, 128 Shenyang Road, Shanghai 200090, China; ∥ Shanghai First Maternity and Infant Hospital, Tongji University School of Medicine, 2699 Gaoke Road, Shanghai 201204, China

**Keywords:** ultrafine particle, neurodevelopment, developmental delay, preschool children, postnatal exposure

## Abstract

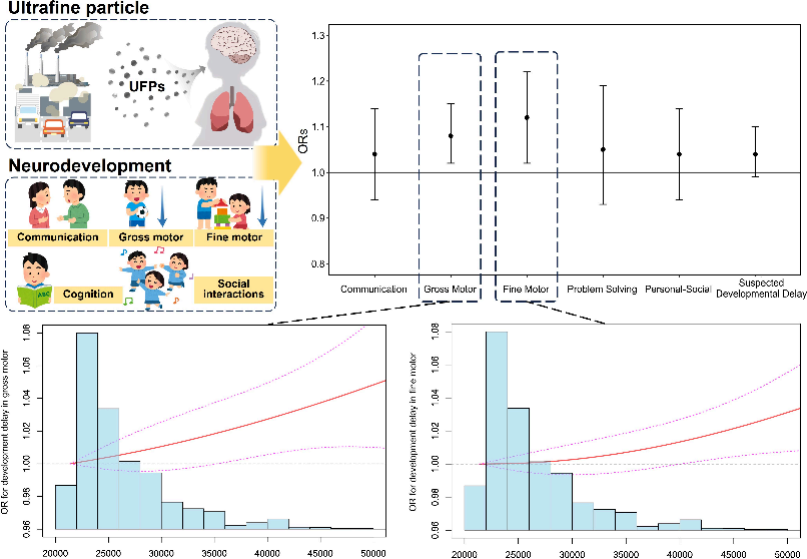

Previous toxicological research has suggested the potential neurotoxicity of ultrafine particulate matter (UFP, particles ≤0.1 μm in diameter). However, evidence from human beings, particularly regarding the neurodevelopmental impacts of UFP, is still limited. We enrolled 11,230 children aged 3–5.5 years from Shanghai, China. Residential UFP exposure was assessed by a land use regression model with a spatial resolution of 50 m. The neurodevelopment of preschoolers was assessed using the Ages & Stages Questionnaires, Third Edition. Generalized linear mixed models were used to examine the associations of UFP exposure with risk of suspected neurodevelopmental delay. For our participants, the median of UFP exposure was 24,478 [interquartile range (IQR): 22,773–27,657] number per cubic centimeter. We observed that each IQR increase in UFP was associated with 8% [odds ratio (OR), 1.08; 95% CI, 1.02–1.15] and 12% (OR, 1.12; 95% CI, 1.02–1.22) higher odds of suspected neurodevelopmental delay in gross and fine motor skills, respectively. These associations show a monotonically upward dose–response manner across overall UFP concentrations. Our findings suggest that UFP exposure during early childhood is associated with an increased risk of neurodevelopmental delay among Chinese preschoolers.

## Introduction

Neurodevelopmental delay in children is characterized by their inability to attain developmental milestones consistent with their peers in cognitive, language, and motor skills development.^1,2^ This issue represents a critical global health concern, particularly in developing countries where factors such as inadequate healthcare access and environmental risks contribute to its higher prevalence.^3,4^ The incidence of neurodevelopmental delays is on the rise, affecting 10–15% of preschool children worldwide.^5^ Such delays can lead to a substantial burden on both childhood and adulthood health. For instance, early global developmental delay may increase the likelihood of developing attention deficit hyperactivity disorder.^6^ Moreover, delays in language or motor development during early childhood can lead to challenges in intelligence, reading comprehension, cognitive skills, behavioral regulation, and academic achievement in later years.^7−11^ Therefore, the early identification of modifiable risk factors and the implementation of targeted interventions are crucial for promoting optimal child neurodevelopment.

Children, due to their developing physiological systems, are particularly considered a vulnerable population to environmental stressors.^12^ Ambient particles, especially those with an aerodynamic diameter less than 2.5 μm (PM_2.5_), have been shown to significantly affect neurodevelopment in children.^13−17^ Previous toxicological studies suggest that ultrafine particles (UFP, particles ≤0.1 μm in diameter) may pose even greater health hazards than PM_2.5_^18^ due to their high surface area to volume ratio, which enables them to adsorb a substantial amount of toxic organic compounds.^19−21^ Moreover, given their nanoscale size,^22,23^ UFPs can penetrate the cardiopulmonary system^21,24,25^ and may even reach the central nervous system.^26−33^ Therefore, understanding the neurological effects of UFPs is of significant public health importance.

Epidemiological studies have linked UFP exposure to neurological disorders in adults and the elderly,^34−39^ but evidence for children, especially in early childhood, remains limited. To the best of our knowledge, only three prior studies in North America^40^ and Western Europe^41,42^ have explored the relationship between UFP exposure and neurodevelopment. Carter et al. reported prenatal exposure to aircraft UFP was associated with autism spectrum disorder (ASD) diagnosis in a cohort of California children at age 5.^40^ Forns et al. and Sunyer et al. linked UFP exposure at school to cognitive development in primary school children (7 to 11 years) in Barcelona, Spain.^41,42^ These existing studies, however, primarily focused on UFP exposure from specific sources (e.g., aircraft emissions) or within specific environments (e.g., schools), limiting the generalizability of their findings to broader exposure contexts. Moreover, the impact of UFPs on specific neurodevelopment domains, such as language and motor skills, has been scarcely investigated. This underscores the urgent need for research in more diverse and high-pollution environments and across a broader spectrum of neurodevelopmental outcomes to gain a better understanding of the effects of UFP exposure.

To address the research gap, we used a subset of data from the Chinese National Cohort of Motor Development (CNCMD) to explore the association of ambient UFP exposure with neurodevelopment and the risk of neurodevelopmental delays in preschool children from Shanghai, China.

## Materials and Methods

### Study Population

The CNCMD is designed to investigate the influence on neurobehavioral development and sleep in young Chinese children. Participant’s recruitment was achieved through stratified cluster sampling, taking account of variables including age, sex, socioeconomic status, and geographic region. More details on the study design of CNCMD can be found elsewhere.^43^ Given the geographic scope of our UFP prediction model, this study focused exclusively on participants from Shanghai, China. Our initial study population consisted of 24,420 preschoolers, recruited from 226 kindergartens and surveyed between September, 2018 and November, 2019. For data analyses, we excluded children aged <3 years or >5.5 years (according to the applicability of our questionnaire for neurodevelopment assessment), who were twins or multiple births, whose maternal conception age was <18 years, and who were lacking covariate information. The process of exclusion, including the numbers excluded at each step, is presented in Figure S1. Following these exclusions, the final study sample comprised 11,230 children. We found the demographic characteristics, UFP concentrations, and prevalence of overall suspected developmental delay (SDD) between the included and initial populations were comparable (Table S1 and Table S2).

Informed consent was obtained from the parents of all participants at the time of enrollment. The confidentiality of participant information was stringently upheld with access restricted to academic research purposes only. The study was approved by the Ethics Committee of the Shanghai First Maternity and Infant Hospital (KS18156).

### Exposure Assessment

For each participant, we collected the detailed residential address after the participant’s birth. UFP concentrations during the study period were measured using a land use regression (LUR) model with a spatial resolution of 50 × 50 m. The UFP monitoring data used for modeling were obtained from 144 fixed monitoring sites across the central urban area and satellite districts in Shanghai, collected over the period between January 8, 2019, and August 31, 2019. This model, which primarily consisted of predictors related to the traffic, buildings, and restaurants, explained most of the spatial variability (69%) in the ambient UFP. More details about the modeling methodology can be found elsewhere.^44^ For each participant, we estimated the annual average concentration of UFP in 2019, using the exposure grid linked to the longitude and latitude of the residential address.

To adjust for potential confounding of other environmental factors, we obtained daily means of PM_2.5_ and nitrogen dioxide (NO_2_) from air quality monitoring stations (http://www.cnemc.cn/) and daily temperature and relative humidity data from meteorological stations (http://data.cma.cn/). The data of the nearest station to a participant’s home address was assigned to the corresponding participant. The annual average concentrations of PM_2.5_ and NO_2_ for each participant were calculated for the year prior to their neurodevelopmental assessment. The timelines of exposure windows of the three air pollutants, along with the neurodevelopmental assessment time period, are illustrated in Figure S2.

### Outcome Measurement

Children’s neurodevelopment was assessed using the Ages & Stages Questionnaires, Third Edition (ASQ-3) between September and November 2019. The ASQ-3 is a parent-reported questionnaire and serves as a screening tool for child development, focusing on individuals aged 1 to 66 months. In this study, we used the Chinese version of the ASQ-3, the specific methods of which have been published elsewhere.^45^ This questionnaire has been validated for assessing the neurodevelopment of children in mainland China, by a comparison with the Gesell Development Scale (specificity = 84.48%, sensitivity = 87.50%, and Cronbach’s alpha coefficient = 0.8).^46^ Furthermore, the feasibility of completing the ASQ-3 by parents or caregivers has also been tested in the Shanghai area, and their assessment scores correlated with those of professionals (*r* = 0.84, *P* < 0.0001).^46^

The ASQ-3 encompasses five domains of neurodevelopment, namely communication, gross motor, fine motor, problem solving, and personal-social skills.^45^ In each domain, six questions about a child’s capability to perform age-appropriate activities and demonstrate developmental milestones are presented, each accompanied by three selectable options (yes = 10 points, sometimes = 5 points, not yet = 0 points). The score for each domain is calculated by summing the scores of all six questions under that domain, and higher scores represent better development in a corresponding area. Children were diagnosed with suspected developmental delay in corresponding dimensions of development if their specific domain of ASQ score was more than 2 standard deviations (SD) below the mean. Additionally, participants were identified as SDD when they had a suspected delay in at least one of the five developmental domains.

### Covariates

Based on extensive knowledge from existing literature and data availability,^14,47,48^ we considered potential covariates as follows. (1) Children’s characteristics: sex (boys, girls), age, body mass index z-score (BMIz), delivery mode (vaginal, cesarean), birth weight, birth year, admission to neonatal intensive care unit (NICU, yes, no), and exclusive breastfeeding (≥6 months or <6 months). (2) Mothers’ physiological status during pregnancy: maternal age at conception, gravidity (primigravida, multigravida), occurrence of maternal complications during pregnancy and delivery (as defined by the International Classification of Diseases, Revision 10, yes, no), and gestational weeks. (3) The socioeconomic status of parents and features of the families: maternal and paternal education (middle school or below, high school, college or above), maternal employment status (employed, unemployed, others), annual household income (≤30,000 CNY, ≤100,000 CNY, >100,000 CNY, unknown), family structure (nuclear household, linear household, joint household), and marital status (first marriage, others).

We used DAGitty’s online tool (www.dagitty.net) to develop a directed acyclic graph to determine the minimal sufficient adjustment set of variables (Figure S3), which includes child’s sex, age, age/sex standardized body mass index (BMIz), maternal age at conception, maternal and paternal education, maternal employment, family income, family structure, marital status, ambient temperature (in natural cubic splines with 6 degrees of freedom), and relative humidity (with 3 degrees of freedom).

### Statistical Analysis

Linear mixed-effect models were used to assess the associations between the UFP exposure and ASQ-3 scores. Generalized linear mixed models (GLMMs) were employed to examine the associations between UFP exposure and the risk of neurodevelopmental delay. Considering a potential correlation within a kindergarten, we included a random intercept of kindergarten in both the linear mixed-effect models and the GLMMs. We fitted a crude model and a model adjusting for the minimal sufficient adjustment set of variables (i.e., the main model).

To evaluate the shape of the exposure–response curves between UFP and risk of SDD, we applied smooth splines with 3 degrees of freedom for UFP concentrations in generalized additive mixed models (GAMMs), which were adjusted for the same covariates with the main model. The exposure–response relationships were examined only for the specific domains with significant associations.

Furthermore, to evaluate the robustness of the results, we additionally adjusted for the delivery mode, exclusive breastfeeding for 6 months, days of pregnancy, gravidity, maternal complications, neonatal ICU admission, birth weight, and birth year of children based on the main model. We also fitted two-pollutant models by including the average concentrations of PM_2.5_ and NO_2_, respectively, based on the main model.

The results of ASQ-3 scores were presented as the changes and their 95% confidence interval (CI) associated with per IQR increases of UFP concentration. The estimates of neurodevelopmental delay were presented as the odds ratio (OR) and 95% CI per IQR increase of UFP exposure. All statistical analyses were performed in R (version 4.2.1) with the “lme 4” and “mgcv” packages for model fitting and the “ggplot2” package for exposure–response curve plotting. The *P* value less than 0.05 in a two-sided test was considered statistically significant.

## Results

### Descriptive Statistics

The characteristics of study participants are summarized in Table 1. In our study, the mean (±SD) of children’s age and BMIz was 4.3 (±0.7) years old and 0.4 (±1.9), respectively. The mean (±SD) of maternal age at conception was 29.0 (±3.9) years old. Approximately 46.6% of the children were delivered by cesarean section, and 47.5% were girls. And 77.6% of mothers and 78.5% of fathers had a high level of education (i.e., college or above). Among our participants,13.5% (*n* = 1519) were identified as having SDD. Compared to the non-SDD group, the SDD group had larger proportion of boys (64.4% vs 50.6%) and a greater percentage of parents with lower education level (i.e., middle school or below; maternal, 15.9% vs 5.7%; paternal, 12.0% vs 5.0%).

**Table 1:** Demographic Characteristics of the Study Participants (*n* = 11,230)^a^

characteristics	SDD (*n* = 1519)	normal (*n* = 9711)	overall (*n* = 11,230)
child age (years)	4.3 (0.7)	4.3 (0.7)	4.3 (0.7)
Sex			
boys	978 (64.4%)	4914 (50.6%)	5892 (52.5%)
girls	541 (35.6%)	4797 (49.4%)	5338 (47.5%)
BMIz	0.6 (2.1)	0.3 (1.9)	0.4 (1.9)
birth weight (g)	3310 (494)	3300 (469)	3300 (472)
Delivery Mode			
vaginal delivery	782 (51.5%)	5213 (53.7%)	5995 (53.4%)
cesarean delivery	737 (48.5%)	4498 (46.3%)	5235 (46.6%)
NICU			
yes	175 (11.5%)	970 (10.0%)	1145 (10.2%)
no	1344 (88.5%)	8741 (90.0%)	10,085 (89.8%)
Exclusive Breastfeeding			
≥6 months	1169 (77.0%)	7819 (80.5%)	8988 (80.0%)
never or <6 months	350 (23.0%)	1892 (19.5%)	2242 (20.0%)
maternal gestational age (years)	28.4 (4.1)	29.1 (3.9)	29.0 (3.9)
Maternal Gravidity			
primigravida	685 (45.1%)	4880 (50.3%)	5565 (49.6%)
multigravida	834 (54.9%)	4831 (49.7%)	5665 (50.4%)
Maternal Education			
middle school or below	241 (15.9%)	554 (5.7%)	795 (7.1%)
high school	367 (24.2%)	1358 (14.0%)	1725 (15.4%)
college or above	911 (60.0%)	7799 (80.3%)	8710 (77.6%)
Paternal Education			
middle school or below	182 (12.0%)	486 (5.0%)	668 (5.9%)
high school	378 (24.9%)	1373 (14.1%)	1751 (15.6%)
college or above	959 (63.1%)	7852 (80.9%)	8811 (78.5%)
Maternal Occupation			
worker or businessman or administrator	1019 (67.1%)	6909 (62.4%)	7928 (70.6%)
unemployed	211 (13.9%)	1035 (10.7%)	1246 (11.1%)
others	289 (19.0%)	1767 (18.2%)	2056 (18.3%)
Marital Status			
first marriage	1426 (93.9%)	9268 (95.4%)	10,694 (95.2%)
others	93 (6.1%)	443 (4.6%)	536 (4.8%)
Family Structure			
nuclear household	925 (60.9%)	5306 (54.6%)	6231 (55.5%)
linear household	543 (35.7%)	4172 (43.0%)	4715 (42.0%)
joint household	51 (3.4%)	233 (2.4%)	284 (2.5%)

a SD, standard deviation; NICU, neonatal intensive care unit; BMIz, body mass index for sex/age z-score; SDD, suspected developmental delay. “SDD” means an abnormal ASQ-3 score in at least one domain. Data are presented as “mean (SD)” or “*n* (%)”.

For our participants, the mean (±SD) scores of five ASQ-3 domains, i.e., communications, gross and fine motor skills, problem solving, and personal-social skills, were 55.2 (±8.8), 51.9 (±10.8), 49.3 (±12.4), 55.0 (±8.9), and 54.5 (±8.4), respectively (Table S3). The mean scores were consistently higher among girls than among boys (Table S3).

The median concentration of UFP exposure was 24,478 number per cubic centimeter (N/cm^3^) (Table S4), which exceeds the guidance (20,000 N/cm^3^) of the World Health Organization Global Air Quality Guidelines in 2021^49^ and is also higher than UFP levels observed in London (13,416 N/cm^3^) and Boston (18,000 N/cm^3^).^50,51^ UFP shows weak correlations with PM_2.5_ and NO_2_ (Spearman correlation coefficient = 0.06 and 0.10) (Table S5). Overall, the exposure level of UFP was found to be relatively higher among the SDD group than the normal (Table S6). The between-group differences reach statistical significance in the domains of gross motor (*P* = 0.004) and fine motor (*P* = 0.03).

### Associations of UFP Exposure with ASQ-3 Scores and SDD Risk

Table 2 summarizes the associations between the UFP and ASQ-3 scores. The crude and adjusted models consistently show robust associations between higher UFP exposure and poorer neurodevelopment (as indicated by lower scores), especially in two domains of gross and fine motor. In the adjusted model, the scores of gross motor and fine motor were 0.22 (95% CI, 0.02–0.42) and 0.30 (95% CI, 0.07–0.52) lower per IQR (4884 N/cm^3^) increase on UFP exposure, respectively.

**Table 2:** Estimates and 95% CIs for the ASQ-3 Scores Associated with Per-Interquartile Range Increase (4884 N/cm^3^) in UFP Exposure among the Preschoolers (*n* = 11,230) in Shanghai, China^a^

	model^b^		adjusted model^c^	
outcome variables	β-coefficients (95% CI)	*P* value	β-coefficients (95% CI)	*P* value
communication	–0.14 (−0.31, 0.03)	0.113	–0.10 (−0.27, 0.06)	0.225
gross motor	**-0.32 (−0.53, −0.11)**	**0.003**	**-0.22 (−0.42, −0.02)**	**0.034**
fine motor	**-0.38 (−0.62, −0.14)**	**0.002**	**-0.30 (−0.52, −0.07)**	**0.010**
problem solving	–0.15 (−0.32, 0.03)	0.097	–0.12 (−0.28, 0.05)	0.159
personal-social	–0.11 (−0.28, 0.05)	0.177	–0.12 (−0.28, 0.03)	0.116

a UFP, ultrafine particle of <0.1 μm in aerodynamic diameter; CI, confidence interval.

b Model: only included UFP and the random contribution of kindergarten in the model.

c Adjusted model: model adjusted for the minimal sufficient adjustment set. Adjusted for child’s sex, age, age/sex standardized body mass index (BMIz), maternal age at conception, maternal and paternal education, maternal employment, family income, family structure, marital status, ambient temperature, relative humidity, and kindergarten.

Table 3 presents the associations between UFP exposure and the risk of suspected neurodevelopmental delay. Our models yielded consistent results, showing that UFP exposure was associated with the higher risk of suspected neurodevelopmental delay in gross and fine motor. In the adjusted model, an IQR increase of UFP concentrations (4884 N/cm^3^) was associated with 8% (OR, 1.08; 95% CI, 1.02–1.15) higher odds of SDD in the gross motor domain and 12% (OR, 1.12; 95% CI, 1.02–1.22) higher odds of SDD in the fine motor domain.

**Table 3:** Adjusted ORs and 95% CIs for Suspected Developmental Delay in Five Developmental Domains and SDD Associated with Per-Interquartile Range Increase (4884 N/cm^3^) in UFP Exposure among the Preschoolers (*n* = 11,230) in Shanghai, China^a^

	model^b^		adjusted model^c^	
outcome variables	OR (95% CI)	*P* value	OR (95% CI)	*P* value
communication	1.05 (0.95, 1.16)	0.330	1.04 (0.94, 1.14)	0.474
gross motor	**1.11 (1.04, 1.18)**	**0.001**	**1.08 (1.02, 1.15)**	**0.010**
fine motor	**1.13 (1.03, 1.25)**	**0.009**	**1.12 (1.02, 1.22)**	**0.019**
problem solving	1.04 (0.92, 1.18)	0.494	1.05 (0.93, 1.19)	0.442
personal-social	1.04 (0.94, 1.14)	0.492	1.04 (0.94, 1.14)	0.435
SDD	1.06 (1.00, 1.11)	0.053	1.04 (0.99, 1.10)	0.125

a UFP, ultrafine particle; OR, odds ratio; CI, confidence interval; SDD, suspected developmental delay. “SDD” means an abnormal ASQ-3 score in at least one domain.

b Model: only included UFP and the random contribution of kindergarten in the model.

c Adjusted model: model adjusted for the minimal sufficient adjustment set. Adjusted for child’s sex, age, age/sex standardized body mass index (BMIz), maternal age at conception, maternal and paternal education, maternal employment, family income, family structure, marital status, ambient temperature, relative humidity, and kindergarten.

**Figure 1:**
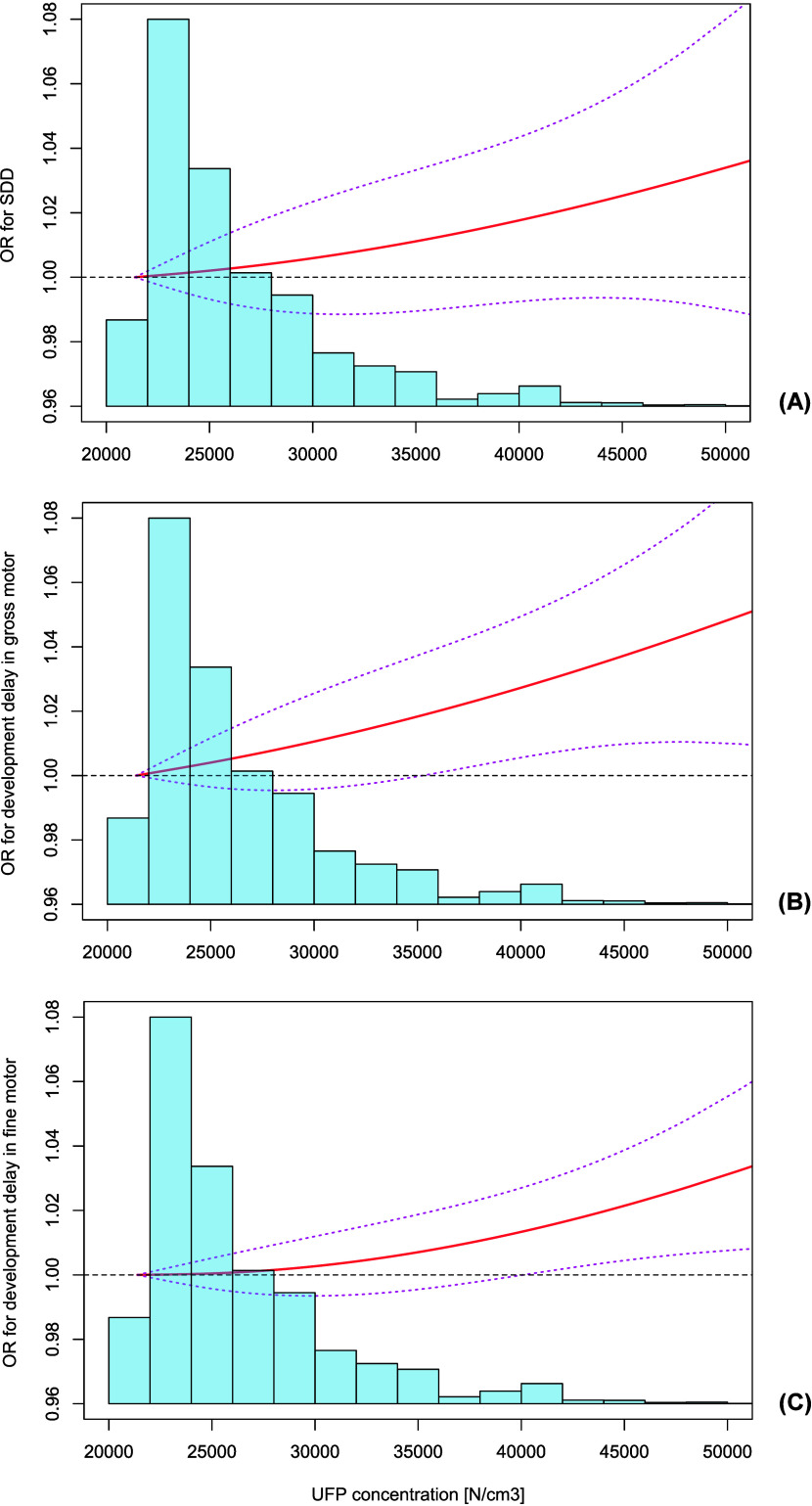
Exposure–response curves between ultrafine particles and the risks of (A) overall, (B) gross motor, and (C) fine motor neurodevelopmental delay in preschool children. Exposure–response curves were fitted by generalized additive mixed models, which were adjusted for the minimal sufficient adjustment set: child’s sex, age, age/sex standardized body mass index (BMIz), maternal age at conception, maternal and paternal education, maternal employment, family income, family structure, marital status, ambient temperature, relative humidity, and the random contribution of kindergarten. UFP, ultrafine particle; ORs, odds ratios; SDD, suspected developmental delay. “SDD” means an abnormal ASQ-3 score in at least one domain.

We further examined the exposure–response relationship curves for UFP concentrations and the likelihood of SDD and motor neurodevelopmental delay (Figure 1). We observed that higher concentrations of UFP exposure were associated with greater odds of overall SDD or SDD in gross and fine motor, with a monotonically upward dose response manner.

### Sensitivity Analysis

Sensitivity analyses showed that the observed associations of UFP exposure with ASQ-3 scores and SDD risk remained robust after adjusting for the delivery mode, exclusive breastfeeding for 6 months, days of pregnancy, gravidity, maternal complications, neonatal ICU admission, birth weight, and birth year of children (Table S7). Additionally, changes in the estimated associations of UFP exposure with ASQ-3 scores and SDD risk after controlling for PM_2.5_ and NO_2_ were minimal (Table 4 and Table 5).

**Table 4:** Estimates and 95% CIs for the ASQ-3 Scores with Per-Interquartile Range Increase (4884 N/cm^3^) in UFP Concentrations by Additionally Adjusting for PM_2.5_ and NO_2_ Based on the Main Model^a^

outcome variables	UFP	+PM_2.5_^b^	+NO_2_^b^
communication	–0.10 (−0.27, 0.06)	–0.10 (−0.27, 0.06)	–0.10 (−0.27, 0.06)
gross motor	–0.22 (−0.42, –0.02)^c^	–0.22 (−0.42, –0.02)^c^	–0.22 (−0.42, –0.02)^c^
fine motor	–0.30 (−0.52, –0.07)^c^	–0.30 (−0.52, –0.07)^c^	–0.30 (−0.52, –0.07)^c^
problem solving	–0.12 (−0.28, 0.05)	–0.12 (−0.28, 0.05)	–0.12 (−0.28, 0.05)
personal-social	–0.12 (−0.28, 0.03)	–0.12 (−0.28, 0.03)	–0.12 (−0.28, 0.03)

a UFP, ultrafine particle; PM_2.5_, fine particulate matter; NO_2_, nitrogen dioxide; CI, confidence interval. Models were adjusted for the minimal sufficient adjustment set: child’s sex, age, age/sex standardized body mass index (BMIz), maternal age at conception, maternal and paternal education, maternal employment, family income, family structure, marital status, ambient temperature, relative humidity, and the random contribution of kindergarten.

b Pollutant exposure of study participants is in the year before competing their neurodevelopmental assessment.

c *P* value < 0.05.

**Table 5:** ORs and 95% CIs for Developmental Delay with Per-Interquartile Range Increase (4884 N/cm^3^) in UFP Concentrations by Additionally Adjusting for PM_2.5_ and NO_2_ Based on the Main Model^a^

outcome variables	UFP	+PM_2.5_^b^	+NO_2_^b^
communication	1.04 (0.94, 1.14)	1.04 (0.94, 1.14)	1.04 (0.94, 1.15)
gross motor	1.08 (1.02, 1.15)^c^	1.08 (1.02, 1.15)^c^	1.08 (1.02, 1.15)^c^
fine motor	1.12 (1.02, 1.22)^c^	1.11 (1.02, 1.22)^c^	1.12 (1.02, 1.22)^c^
problem solving	1.05 (0.93, 1.19)	1.05 (0.93, 1.19)	1.05 (0.92, 1.19)
personal-social	1.04 (0.94, 1.14)	1.04 (0.94, 1.14)	1.04 (0.94, 1.15)
SDD	1.04 (0.99, 1.10)	1.04 (0.99, 1.10)	1.04 (0.99, 1.10)

a UFP, ultrafine particle; OR, odds ratio; CI, confidence interval; SDD, suspected developmental delay. “SDD” means an abnormal ASQ-3 score in at least one domain. PM_2.5_, fine particulate matter; NO_2_, nitrogen dioxide. Models were adjusted for the minimal sufficient adjustment set: child’s sex, age, age/sex standardized body mass index (BMIz), maternal age at conception, maternal and paternal education, maternal employment, family income, family structure, marital status, ambient temperature, relative humidity, and the random contribution of kindergarten.

b Pollutant exposure of study participants is in the year before competing their neurodevelopmental assessment.

c *P* value < 0.05.

## Discussion

This study investigated the association between ambient UFP exposure and neurodevelopmental delay in a cohort of 11,230 preschool children aged 3 to 5.5 years in Shanghai, China. Our findings indicate that early life exposure to UFP was associated with reduced neurodevelopmental performance and a higher risk of neurodevelopmental delays. This aligns with previous research, such as a study in Barcelona, Spain, which reported the adverse impact of UFP on the cognitive development in children aged 7 to 10 years,^42^ with the effect persisting over 3.5 years.^41^ Another study suggested an association between maternal UFP exposure and an increased risk of ASD in children under the age of 5.^40^ These studies, though varying in age focus (from preschool to primary school children) and exposure periods (prenatal vs postnatal), consistently demonstrate UFP’s detrimental effects on child neurodevelopment.

Our research contributes to this body of knowledge by evaluating the impact of UFP exposure across five neurodevelopmental domains: communication, gross motor, fine motor, problem solving, and personal-social skills. We found that UFP exposure may particularly impact motor development in preschool children. This is evidenced by a distinct exposure–response curve showing an increased risk of developmental delay in motor skills with rising UFP concentration. Given that the association between UFP exposure and motor development has not been extensively studied, it could be challenging to make direct comparisons with the existing literature. Nonetheless, our observations are somewhat supported by research on larger particulate matter. For instance, a recent meta-analysis indicated a 1.39 (95% CI: 0.40–2.38, *P* = 0.006) lower in scores of gross motor development per 1 μg/m^3^ increase in PM_2.5_ exposure in children.^52^ In addition, a comprehensive study in China highlighted a significant association between PM_2.5_ exposure from birth to 36 months of the offspring and the development of general coordination (−0.09; 95% CI, −0.14, −0.04) and control during movement (−0.08; 95% CI, −0.13, −0.03) in preschool children.^14^ The results from our two-pollutant (UFP and PM_2.5_ or UFP and NO_2_) models, the prevailing approach to adjust for air pollutants,^53^ might suggest an independent association between UFP exposure and motor development in preschoolers.

During early childhood, rapid human brain development creates sensitive and vulnerable windows to environmental exposures.^54^ Although the physiological mechanism underlying neurodevelopmental toxicity due to UFP exposure remains unclear, several potential pathways have been proposed. UFP may access the brain by penetrating alveoli and entering the bloodstream, subsequently breaching the blood–brain barrier.^25,55^ Alternatively, UFP might directly migrate through the olfactory nerves.^18,22^ A previous study detected combustion-derived particles in the brains of young individuals^22^ and documented the potential neurobiological relevance of nanoparticles. Once UFP infiltrates the brain, it may subsequently induce inflammatory responses in specific areas such as the cerebral cortex,^56−58^ hippocampus,^57−59^ and cerebellum,^60^ thus representing a primary mechanism of neurodevelopmental toxicity.^61^ Particularly, the inflammatory response noted in the cerebellum, a crucial center for coordination and motor development, provides clues about the potential mechanisms underlying the observed association between UFP exposure and motor development.^60^ Additionally, UFP may provoke oxidative stress, disrupt developmentally vital neurotransmitters, or lead to lateral ventricle dilation.^30,62^

Our study has several strengths. First, this is the first attempt to explore the impact of childhood UFP exposure on specific domains of neurodevelopment in preschool children. Second, we employed a modeling approach with high spatial resolution (50m × 50 m) for assessing UFP exposure. This model captures the majority of UFP variation (∼70%) in the Shanghai region, facilitating the inclusion of a substantial sample size in the exposure assessment. Third, our study rigorously evaluated outcomes and meticulously collected data on established confounding variables, ensuring the high quality of the data set.

However, our study also has several limitations. First, the possibility of exposure measurement errors cannot be entirely discounted, as we did not account for indoor air pollution or children’s individual time-activity. Moreover, the LUR model was developed using monitoring data collected from a single year (2019), and approximately 30% variability remains unexplained in our LUR model, which might lead to unmeasured errors in UFP concentration predictions. Second, due to the cross-sectional design of this study, the ambiguous temporal sequence may limit the causal inferences between UFP exposure and developmental delay. Third, the selection bias cannot be fully eliminated due to the slight differences between the included and initial participants. Additionally, ASQ-3 is a parent-reported questionnaire, which may introduce certain reporting bias. Lastly, a potential for residual confounding exists, as certain factors, such as secondhand smoking, were not adjusted due to a lack of available information. These unaccounted variables may exert an influence on the observed associations and should be considered when interpreting the study’s findings.

## Conclusion

In conclusion, this study identified significant associations between childhood UFP exposure and lower ASQ-3 scores and a higher risk of neurodevelopmental delays, particularly in the domains of gross and fine motor development among preschoolers in Shanghai, China. This research may add evidence to the impact of UFP on childhood neurodevelopment.

## Supporting Information Available

Flowchart of the study population; timeline of the environmental and outcome data collection; directed acyclic graph of neurodevelopmental delay and UFP exposure; summary characteristics of included and initial participants; descriptive statistics of the UFP concentration and the prevalence of SDD in the study samples; descriptive statistics of ASQ-3 questionnaire scores; descriptive statistics of ambient pollutants exposure; correlation between air pollutants; mean UFP concentrations between normal and suspected developmental delay groups; and regression results of model adjusted for all the covariates (PDF)
