# Supplementary material for: Association between Ambient Ultrafine Particles and Neurodevelopmental Delay in Preschoolers in Shanghai, China
Source: Environ Health (Wash). 2024 Oct 1;3(1):102–10. doi: 10.1021/envhealth.4c00102 (PMC11744390; doi:10.1021/envhealth.4c00102)
Supplement: Supplementary file 1 — eh4c00102_si_001.pdf [file eh4c00102_si_001.pdf]

Supporting Information

Association between ambient ultrafine particle and neurodevelopmental delay in preschoolers in Shanghai, China

Mengxun Rong[1], Yang Shen[1], Yihui Ge[1], Wenchong Du[2], Haidong Kan[1], Jing Cai[1], Yan Zhao[3*] and Jing Hua[4*]*

[1] Department of Environmental Health, School of Public Health, Fudan University, 130 Dong-An Road, Shanghai 200032, China

[2] Department of Psychology, Nottingham Trent University, Burton Street, Nottingham, NG1 4BU, UK

[3] Hospital of Obstetrics and Gynecology, Shanghai Medical School, Fudan University, 128 Shenyang Road, Shanghai 200080, China

[4] Shanghai First Maternity and Infant Hospital, Tongji University School of Medicine, 2699 Gaoke Road, Shanghai 201204, China

***** Jing Cai's E-mail: jingcai@fudan.edu.cn

Yan Zhao's E-mail: zy861201@163.com

Jing Hua's E-mail: jinghua@tongji.edu.cn

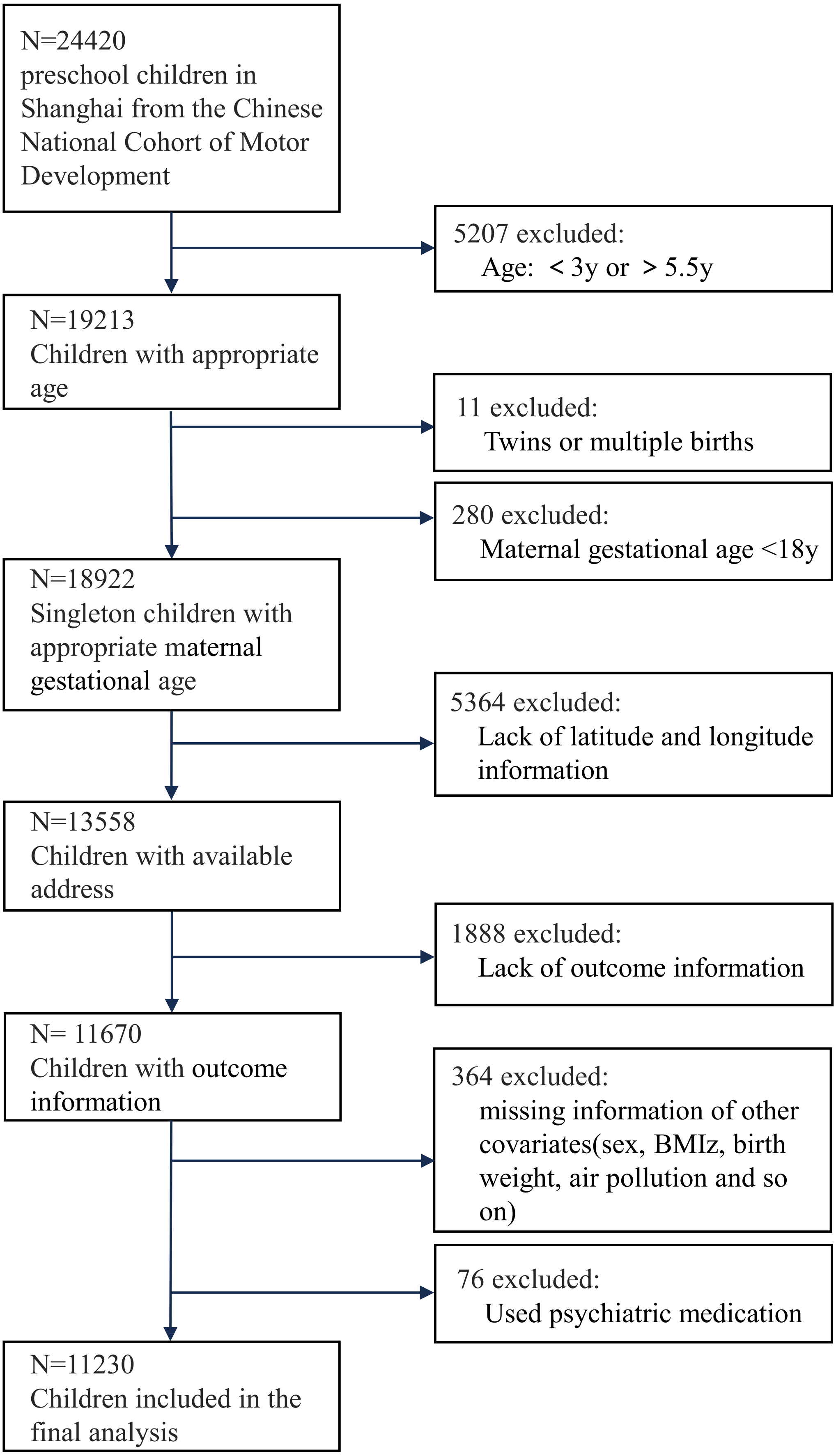

Figure S1. Flow chart of the study population.

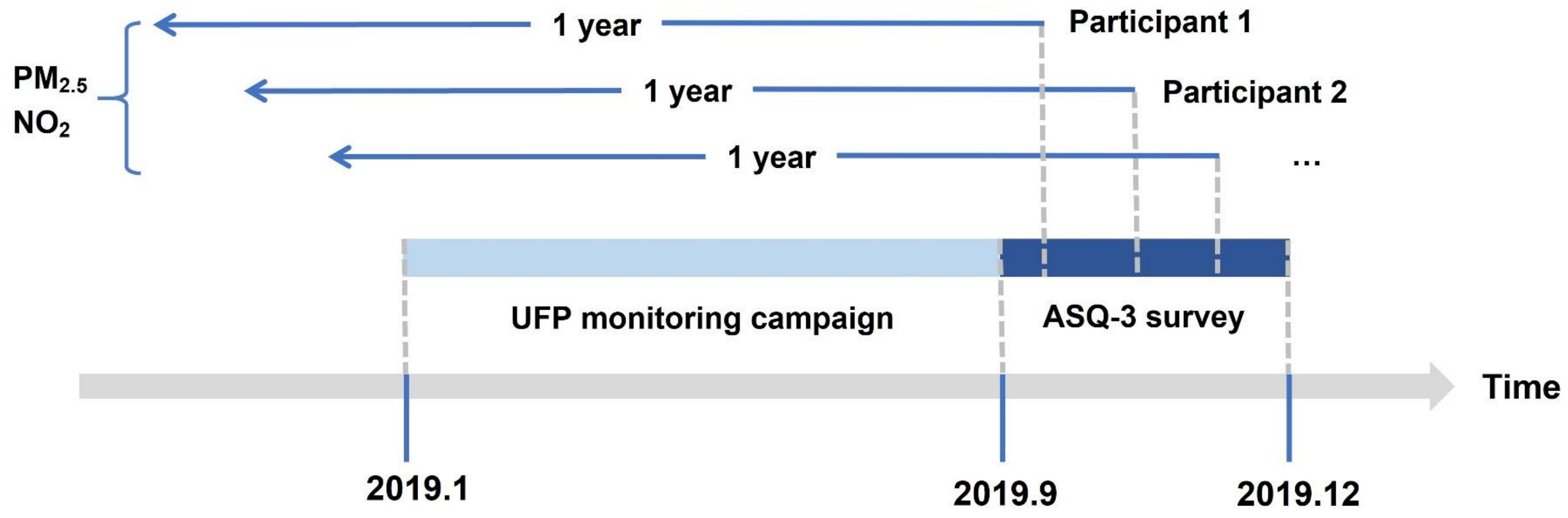

Figure S2. The timeline of the environmental and outcome data collection.

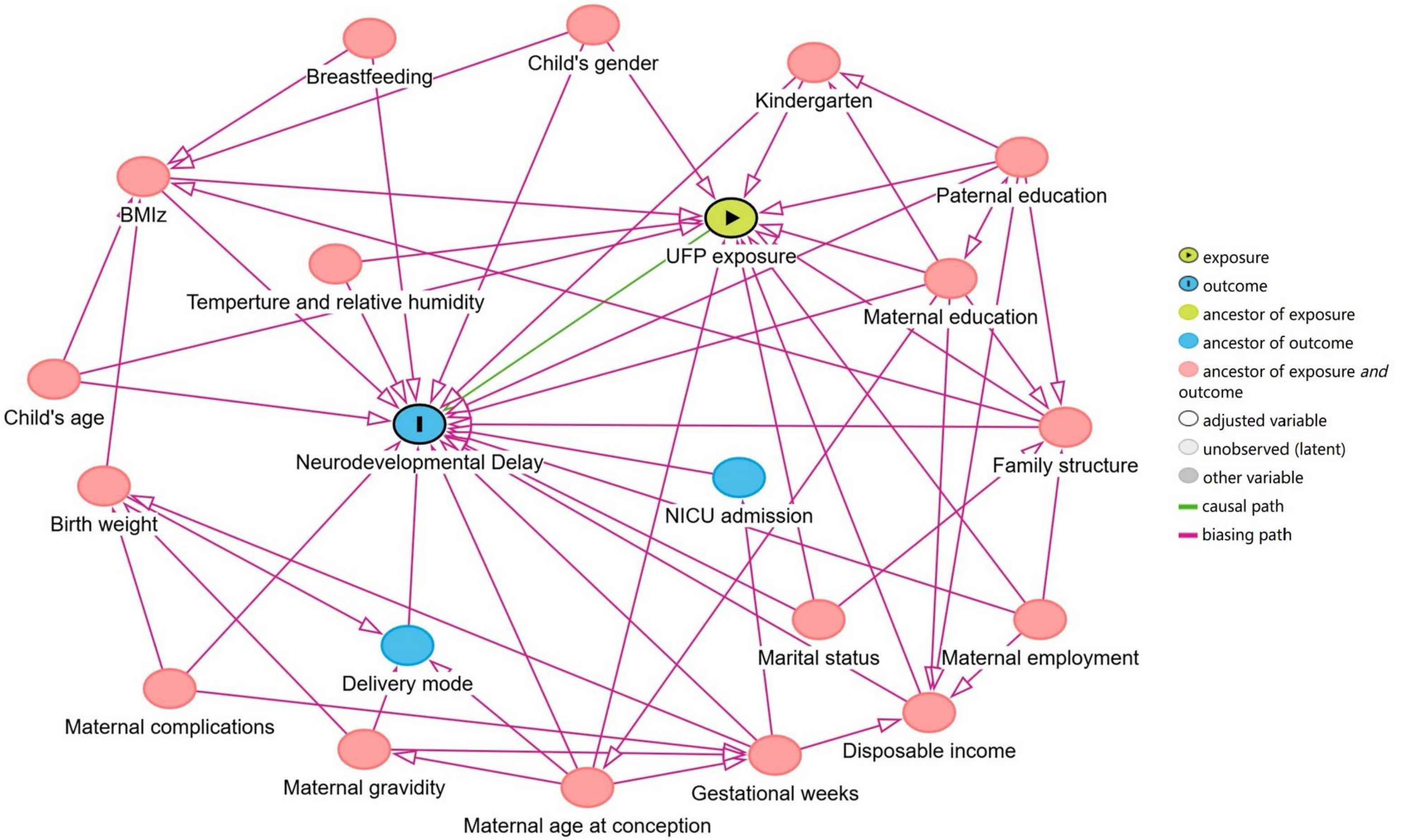

Figure S3. Directed acyclic graph (DAG) of neurodevelopmental delay and UFP exposure. The nodes and arrows represent variables and the causal associations between them, respectively.

Table S1. Summary characteristics of included and initial participants

Characteristics	Mean ± SD or n (%)	
	Included participants (n = 11230)	Initial participants (n = 24420)
**Child age (years)**	4.3 (0.7)	4.6 (1.0)
**Sex**		
Boys	5892 (52.5%)	13012 (53.3%)
Girls	5338 (47.5%)	11407 (46.7%)
**BMIz**	0.4 (1.9)	0.4 (1.9)
**Birth weight (g)**	3300 (472)	3200 (607)
**Delivery mode**		
Vaginal delivery	5995 (53.4%)	12091 (49.5%)
Cesarean delivery	5235 (46.6%)	12329 (50.5%)
**NICU**		
Yes	1145 (10.2%)	2420 (9.9%)
No	10085 (89.8%)	22000 (90.1%)
**Exclusive breastfeeding**		
≥ 6 months	8988 (80.0%)	18994 (78.3%)
Never or < 6 months	2242 (20.0%)	5263 (21.7%)
**Maternal gestational age (years)**	29.0 (3.9)	28.4 (4.1)
**Maternal gravidity**		
Primigravida	5565 (49.6%)	7690 (49.3%)
Multigravida	5665 (50.4%)	7923 (50.7%)
**Maternal education**		
Middle school or below	795 (7.1%)	2361 (9.7%)
High school	1725 (15.4%)	4243 (17.4%)

College or above	8710 (77.6%)	17816 (73.0%)
**Paternal education**		
Middle school or below	668 (5.9%)	1947 (8.0%)
High school	1751 (15.6%)	4362 (17.9%)
College or above	8811 (78.5%)	18111 (74.2%)
**Maternal occupation**		
Worker or businessman or administrator	7928 (70.6%)	17335 (71.0%)
Unemployed	1246 (11.1%)	2635 (10.8%)
Others	2056 (18.3%)	4450 (18.2%)
**Marital status**		
First marriage	10694 (95.2%)	23058 (94.4%)
Others	536 (4.8%)	1361 (5.6%)
**Family structure**		
Nuclear household	6231 (55.5%)	13869 (56.8%)
Linear household	4715 (42.0%)	9926 (40.6%)
Joint household	284 (2.5%)	625 (2.6%)

Note: SD, standard deviation; BMIz, body mass index for sex/age z-score; NICU, neonatal intensive care unit.
The percentage of missing was calculated as the percentage of the number of the information missing participants relative to the number of excluded participants.

Table S2. The UFP concentration and the prevalence of SDD in the study samples

Characteristic	Total number	UFP [N/cm^3]	SDD
		Median [P_{25}, P_{75}]	n (%)
**Total participants**	**24420**	**24593 [22849, 27870]**	**2190 (14.0%)**
Children's aged < 3y or > 5.5y	5207	24900 [22951, 28541]	306 (11.9%)
Twins or multiple births	11	24898 [22876, 30062]	3 (37.5%)
Maternal gestational age < 18y	280	24416 [22784, 27450]	29 (15.1%)
Lack of exposure information	5364	-	231 (19.7%)
Lack of outcome information	1888	25266 [22992, 27785]	-
Missing information of covariates	364	24429 [22928, 27450]	88 (24.2%)
Used psychiatric medication	76	24478 [22606, 28143]	15 (19.7%)
**Included participants**	**11230**	**24478 [22773, 27657]**	**1519 (13.5%)**

Note: UFP, ultrafine particle; SDD, suspected developmental delay, "SDD" means an abnormal ASQ-3 score in at least one domain; P_{25}, percentile 25; P_{75}, percentile 75.

Table S3. Descriptive statistics of ASQ-3 scores of participants in five subscales

Outcome Variables	Total (N=11230)	Boys (n=5892)	Girls (n=5338)
	Mean ± SD	Mean ± SD	Mean ± SD
**Communication**	55.2 ± 8.8	54.6 ± 9.4	55.9 ± 8.0
**Gross Motor**	51.9 ± 10.8	51.3 ± 11.2	52.6 ± 10.2
**Fine Motor**	49.3 ± 12.4	47.4 ± 13.3	51.4 ± 11.1
**Problem Solving**	55.0 ± 8.9	54.4 ± 9.3	55.7 ± 8.3
**Personal-Social**	54.5 ± 8.4	53.3 ± 9.0	55.8 ± 7.4

Note: SD, standard deviation.

Table S4. Descriptive statistics of ambient pollutants exposure

Pollutants	Parameters							
	**Mean**	**SD**	**Min**	**Max**	**P_{25}**	**P_{50}**	**P_{75}**	**IQR**
**UFP [N/cm^3]**	26,000	5,021	15,333	82,940	22,773	24,478	27,657	4,884
**$PM_{2.5}$[a] [µg/m^3]**	23	5	18	55	19	19	26	7
**NO_2[a] [µg/m^3]**	35	17	9	74	24	26	51	27

Note: SD, standard deviation; Min, minimum; Max, maximum; P_{25}, percentile 25; P_{50}, percentile 50; P_{75}, percentile 75; IQR, interquartile range; UFP, ultrafine particle; N/cm^3, the number per cubic centimeter; $PM_{2.5}$, fine particulate matter; NO_2, nitrogen dioxide.

[a] means daily averages of pollutant concentrations during the year before our investigation.

Table S5. Spearman correlation coefficients between air pollutant concentrations

	UFP	$PM_{2.5}$	NO_2
**UFP**	1.00		
**$PM_{2.5}$**	0.06[a]	1.00	
**NO_2**	0.10[a]	0.52[a]	1.00

Note: UFP, ultrafine particle; $PM_{2.5}$, fine particulate matter; NO_2, nitrogen dioxide.
[a] *P* value < 0.05.

Table S6. Mean UFP concentrations between normal and suspected developmental delay groups

UFP [N/cm^3]	Mean ± SD (Number of subjects)		
	Normal	Developmental delay	*P*
**Communication**	25,991±5,001 (10,841)	26,275±5,552 (389)	0.27
**Gross Motor**	**25,951±4,945 (10,187)**	**26,484±5,698 (1,043)**	**<0.01**
**Fine Motor**	**25,978±4,977 (10,851)**	**26,666±6,123 (379)**	**0.03**
**Problem Solving**	25,999±5,010 (10,976)	26,101±5,495 (254)	0.75
**Personal-Social**	25,995±5,010 (10,804)	26,148±5,315 (426)	0.54
**SDD**	**25,961±4,949 (9,711)**	**26,258±5,458 (1,519)**	**0.04**

Note: UFP, ultrafine particle; SDD, suspected developmental delay, "SDD" means an abnormal ASQ-3 score in at least one domain. *P* values were obtained by Student t/t' test.

Table S7. Estimates (95% CIs) for the ASQ-3 scores and ORs (95% CIs) for the risk of suspected developmental delays associated with per-interquartile range increase (4884 N/cm³) in UFP exposure among the preschoolers in Shanghai

Outcome Variables	Model adjusted for all the covariates			
	β-Coefficients (95%CI)	*P*-value	OR (95%CI)	*P*-value
**Communication**	-0.10 (-0.26, 0.07)	0.242	1.04 (0.94, 1.14)	0.473
**Gross Motor**	**-0.22 (-0.42, -0.02)**	**0.029**	**1.08 (1.02, 1.15)**	**0.009**
**Fine Motor**	**-0.29 (-0.51, -0.06)**	**0.013**	**1.10 (1.01, 1.21)**	**0.036**
**Problem Solving**	-0.11 (-0.27, 0.06)	0.205	1.04 (0.92, 1.18)	0.512
**Personal-Social**	-0.12 (-0.28, 0.03)	0.121	1.03 (0.93, 1.13)	0.586
**SDD**	-	-	1.04 (0.99, 1.10)	0.130

Note: UFP, ultrafine particle; OR, odds ratio; CI, confidence interval; SDD, suspected developmental delay, "SDD" means an abnormal ASQ-3 score in at least one domain. Model adjusted for all covariates: further adjusted for delivery mode, exclusive breastfeeding for 6 months, days of pregnancy, gravidity, maternal complications, neonatal ICU admission, birth weight and birth year of children based on the adjusted model.
